# Identification of Clinical and Laboratory Parameters Associated with the Development of Acute Chest Syndrome during Vaso-Occlusive Episodes in Children with Sickle Cell Disease: A Preliminary Step before Assessing Specific and Early Treatment Strategies

**DOI:** 10.3390/jcm8111839

**Published:** 2019-11-01

**Authors:** Fouad Madhi, Annie Kamdem, Camille Jung, Adele Carlier-Gonod, Sandra Biscardi, Jeremy Busca, Cecile Arnaud, Isabelle Hau, David Narbey, Ralph Epaud, Corinne Pondarre

**Affiliations:** 1 Service de Pédiatrie Générale, Centre Hospitalier Intercommunal de Créteil, 40 avenue de Verdun, 94000 Créteil, France; adele.carlier-gonod@chicreteil.fr (A.C.-G.); isabelle.hau@chicreteil.fr (I.H.); ralph.epaud@chicreteil.fr (R.E.); 2 Service de pédiatrie, Centre de référence de la Drépanocytose, Centre Hospitalier Intercommunal de Créteil, 94000 Créteil, France; annie.kamdem@chicreteil.fr (A.K.); buscajeremy@gmail.com (J.B.); cecile.arnaud@chicreteil.fr (C.A.); corinne.pondarre@chicreteil.fr (C.P.); 3 Centre de Recherche Clinique, Centre Hospitalier Intercommunal de Créteil, 40 avenue de Verdun, 94000 Créteil, France; camille.jung@chicreteil.fr; 4 Service des Urgences Pédiatriques, Centre Hospitalier Intercommunal de Créteil, 40 avenue de Verdun, 94000 Créteil, France; sandra.biscardi@chicreteil.fr; 5 Centre D’appui Pour la Prévention des Infections Associées aux Soins Auvergne-Rhône-Alpes, Hôpital Henry Gabrielle, 20 route de Vourles, 69230 Saint-Genis-Laval, France; david.narbey@chu-lyon.fr; 6 INSERM Unité 955, Paris XII University, 94000 Créteil, France

**Keywords:** acute chest syndrome, vaso-occlusive episodes, sickle cell disease, associated factors

## Abstract

This prospective observational study sought to ascertain clinical and laboratory parameters associated with the development of acute chest syndrome (ACS) during vaso-occlusive episodes (VOE) in children with sickle cell disease (SCD). It was performed at the pediatric department of the university Intercommunal Créteil hospital. All children with SCD (all sickle genotypes) consecutively admitted from November 2013 to December 2016 for painful VOEs and no evidence of ACS were included. Clinical and laboratory parameters collected at admission and within 48 h after admission were compared for children in whom ACS developed or not. Variables that were statistically significant on univariate analysis or considered to be clinically relevant were included in a multivariable model to ascertain the risk factors associated with the development of ACS during a VOE. The variables retained in the multivariate model were used to construct a predictive score for ACS. For each included child and during the study period, only data from the first VOE and/or the first ACS were analyzed. Among 191 hospitalizations for painful VOEs, for 176 children with SCD, ACS developed in 35 during hospitalization. Mean hospital stay was longer for children with ACS versus VOEs alone (7.6 (±2.3) vs. 3.3 (±1.8) days, *p* < 0.0001), and all children with ACS versus 28/156 (17.9%) with VOEs alone received red blood cell transfusion (*p* < 0.0001). The multivariate model retained pain score (≥9/10), pain localization (abdominal or spinal pain or involving more than two limbs), and high reticulocyte (≥260 × 10^9^/L) and neutrophil (>10 × 10^9^/L) counts, at admission, as independently associated with ACS development. The area under the receiver operating characteristic curve for the ACS predictive score was 0.82 (95% CI: 0.74–0.89), and the negative predictive value was 97.7%. The evolution profiles during the first 48 h differed between children with ACS and VOEs alone, with a more rapid decline of pain score and leucocytosis in children with VOEs. Clinical and laboratory measurements at admission may be simple parameters to identify children with increased risk of ACS development during VOEs and to facilitate early diagnosis of this respiratory complication. Also, the persistent elevation of leukocyte count on day 2 may be considered a sign of evolving ACS.

## 1. Introduction

Sickle cell disease (SCD) is an increasing genetic hemoglobinopathy in France, with a persistent high morbidity among children [1]. Vaso-occlusive episodes (VOEs), also known as acute severe pain episodes, are the leading cause of hospitalization among children with SCD [1]. In children admitted for VOE, the main severe complication is the secondary development of acute chest syndrome (ACS), an acute clinical condition of SCD defined by fever and/or respiratory symptoms and accompanied by new pulmonary infiltrate on a chest X-ray [2]. Children usually experience respiratory distress, appearing within a few days after admission, with preferential lower-lobe involvement. ACS may have a severe clinical course and can progress rapidly from mild hypoxemia to respiratory failure and death [3,4,5,6]. Therefore, increasing the level of clinical suspicion to allow early recognition is essential. ACS is also associated with additional significant morbidity, including prolonged respiratory distress, pain, and hospitalization [7,8].

Despite the well-described association between ACS and admissions for VOE, the epidemiology and risk factors of ACS complication during VOE have not been elucidated. Indeed, the two landmark studies investigating the natural history of SCD, analyzed the data on ACS episodes in general, irrespective of the etiology and did not focus on ACS episodes that developed over the clinical course of VOE [3,4].

In children hospitalized with VOE, the secondary development of ACS has a complex and interrelated pathophysiology, including fat embolism from bone-marrow necrosis and the release of fat emboli; pulmonary microvascular occlusion and infarction; and hypoventilation/atelectasis resulting from splinting (abdominal, rib, or vertebral infarction) and/or opiate narcosis [3,4,5,6,9]. In addition, nitric oxide consumption by cell-free plasma hemoglobin (Hb) during acute hemolysis can lead to pulmonary vasoconstriction and acute pulmonary hypertension [10]. Hemolysis can contribute to acute lung injury via the release of free heme into plasma, which in turn activates the innate immune response [11]. An infective cause is common in children, but this group of ACS differs from ACS episodes that develop over the clinical course of VOEs, with children at admission usually presenting fever, cough, and wheezing; isolated upper- or middle-lobe involvement; and usually showing a less severe course [6].

Nearly half of all ACS episodes occur after 1–3 days after admission for acute severe pain [4,12], which suggests that the VOE may be a prodromal event for the development of ACS. However, only incentive spirometry, performed aggressively in patients hospitalized with VOEs, has been shown to reduce the risk of evolution to ACS [13]. Moreover, once ACS is diagnosed, the treatment is mostly supportive and non-specific (adequate hydration, oxygenation, non-invasive or invasive ventilation if needed, analgesia, antibiotics or antivirals, and bronchodilators). Red blood cell (RBC) transfusion, the only “specific” treatment, is recommended by most experts but increasingly feared by others [14,15]. Its beneficial effect has been suggested by a few studies [16,17,18,19], but no strong comparative randomized studies have been conducted so far [20].

Several risk factors have been found associated with increased incidence of ACS in children, including young age, severe sickle genotypes (HbSS, HbSβ^0^), low fetal Hb level, high steady-state Hb levels, high steady-state leukocyte count, history of asthma, and history of ACS [3,4,5,6,21,22]. However, in the setting of hospitalization for VOEs, little is known about the factors associated with ACS complication, and determining the children with VOEs in whom ACS will develop has remained challenging. The aim of our study was to identify clinical and laboratory parameters associated with ACS development during VOE in order to improve inpatient management in children with VOEs. In addition, this study was conducted as a preliminary step before assessing specific and early treatment strategies to reduce ACS complications.

## 2. Materials and Methods

### 2.1. Study Population

This was a prospective non-interventional study conducted in the pediatric department of the university Intercommunal Créteil hospital, a referral center for SCD. All children < 18 years old with SCD, regardless of sickle genotype (HbSS, SC, SDpunjab Sβ±, or Sβ0-thalassemia) or history of prior ACS or asthma, consecutively admitted from November 2013 to December 2016 from our emergency department for painful VOEs with no evidence of ACS were included. The inclusion criteria were children presenting VOE with normal chest x-ray at admission, with or without fever, treated or not, with hydroxyurea. The exclusion criteria were children presenting to emergency department with obvious or minimal signs of ACS (isolated respiratory symptoms or abnormal lung auscultation, or hypoxia) such as infants with viral bronchiolitis, or with radiological abnormalities suggestive of an ACS, and children receiving a regular transfusion program or after bone marrow transplantation.

### 2.2. Management at the Hospital

In accordance with French national guidelines, RBC transfusion was indicated as first-line treatment, as soon as ACS was diagnosed (either simple or exchange transfusion, depending on the Hb level, and the clinical status) [23]. Other indications for RBC transfusion were severe episodes with pain refractory to morphine, or acute anemia with Hb level < 6 g/dL or decreased by more than 2 g/dL from steady state. According to our local written protocols, advised treatments included use of bedside incentive spirometry (every 4 h, with the assistance of the nurse or the parents), oxygen, intravenous fluids (1.5 L/m^2^), and pain relief. The selection of pain medication (intravenous nalbuphine or morphine using patient-controlled analgesia) was the choice of the admitting pediatrician, but morphine use was suggested for pain score > 6/10. Antibiotic therapy consisting of third-generation cephalosporin and macrolides was prescribed as soon as ACS was diagnosed. Children with VOEs received ceftriaxone only if they had fever during hospitalization. Chest X-ray was performed for every child admitted for a VOE, regardless of the localization of the pain or any respiratory symptoms. Pulse oximetry monitoring was mandatory for all patients admitted with a VOE and oxygen saturation (SpO2) was measured at least every 4 h on room air to enhance the sensitivity and specificity of the test to detect significant hypoxia.

### 2.3. Definitions

A VOE was defined as hospitalization for pain requiring parenteral narcotics that was not attributable to other causes [2]. ACS was defined as any respiratory symptom or thoracic pain or fever associated with a new pulmonary infiltrate on chest X-ray [2]. Hypoxia (SpO2 ≤ 94% on air) was also considered a sign of evolving ACS. History of asthma was defined as having both a reported pediatrician diagnosis of asthma and a history of prescription of an asthma medication (controller and/or rescue medication).

### 2.4. Collected Data

Steady-state laboratory parameters (leukocyte, neutrophil, reticulocyte, and platelet counts; Hb level), HbF, bilirubin, LDH, and glucose-6-phosphate dehydrogenase (G6PD) levels refer to data obtained between 12 to 36 months of age and a minimum of three months away from a transfusion, one month from a painful episode, and before intensive therapy.

Clinical data such as temperature, blood pressure (BP), heart rate (HR), respiratory rate (RR), faces pain score (FPS) [24] using the faces pain scale-revised (FPS-R), and SpO2 on air were collected at least every 4 h.

The following laboratory parameters were collected at admission and 48 h after admission: leukocyte, neutrophil, reticulocyte, and platelet counts; Hb, lactate dehydrogenase (LDH), C-reactive protein (CRP), and fibrinogen levels; fetal hemoglobin (HbF) and hemoglobin S (HbS) levels; and total bilirubin, alanine transaminase (ALT), and aspartate transaminase (AST) levels.

Chest X-ray was performed at admission to exclude ACS and repeated during hospitalization, in case of clinical suspicion of ACS development (respiratory symptoms/hypoxia, thoracic pain, or fever).

### 2.5. Ethics

Parental oral informed consent was obtained in accordance with the Declaration of Helsinki. All data were prospectively and systematically collected in a clinical database. Use of the database was approved for this project by the Créteil Institutional Review Board and by the French National Data Protection Commission (CNIL, no. 2069568).

### 2.6. Statistical Analysis

For each included child and during the study period, only data from the first VOE and/or the first ACS were analyzed. If a child was admitted several times but was not classified in the same group, the first hospitalization in each group was counted as a different case.

The VOE group refers to the episodes limited to VOE, whereas the ACS group refers to ACS episodes developing after admission for a VOE. Data are expressed as mean ± SD or median and interquartile range (IQR) for continuous variables, and number (%) for categorical variables. Student *t* test was used to compare the distribution of variables measured. Chi-square test or Fischer’s exact test was used to compare categorical variables. Logistic regression analysis was performed to identify risk factors of ACS, with VOE characteristics as explanatory variables. Quantitative variables were transformed into binary variables on the basis of cutoffs determined by receiver operating characteristic (ROC) curve analysis. We performed stepwise logistic regression for the multivariable analysis. The probability threshold for covariate entry into the model was *p* < 0.20 and for covariate removal, *p* < 0.10. Data are presented as adjusted odds ratios (ORs) with 95% confidence intervals (CIs). Goodness-of-fit was assessed by the Hosmer–Lemeshow test, and the improvement in amount of variability explained by the model was assessed by calculating MacFaden’s R2 and Nagelkerke’s R2. We assessed the discriminant power of the model by calculating the c-statistic, corresponding to the area under the ROC curve [25].

A predictive risk score for ACS occurrence was created by rounding the OR for each significant variable to the nearest integer. The final score is the sum of the points accorded for all these parameters. The performance of the predictive score was assessed by plotting ROC curves and calculating the c-statistic and its 95% CI. Total precision, Youden index, positive and negative predictive values (PPV, NPV) and Matthews correlation coefficient were used to determine two cutoffs to maximize measurement precision and minimize classification errors. Resampling was used for internal validation of the score, with the bootstrapping method providing the least biased estimate of the c-statistic [11]. STATA13/SE (StataCorp, College Station, TX, USA) was used for descriptive statistical analyses, and SAS 9.4 (SAS Inst., Cary, NC, USA) was used for logistic regression and predictive score analyses. *p* < 0.05 was considered statistically significant. 

## 3. Results

Over the three-year study period, there were 451 hospital admissions for painful VOEs, and 37 preceded ACS (8.2%). Because only data from the first VOE and/or the first ACS were analyzed and all subsequent episodes were excluded from the analysis, our final study included 191 hospitalizations for VOEs in 176 children. Fifteen children were classified in both the VOE and ACS groups, each admission counting as a different case for the evaluation of risk factors for ACS development.

### 3.1. Patient History and Laboratory Parameters at Steady State According to the Development of ASC after Hospitalization for VOEs in the 176 Children

The patient history and laboratory parameters at steady state according to the development of ACS are summarized in Table 1. The mean age of children was 9 (±5.2) years. ACS developed in 35 children during a VOE. Age, sex, history of asthma, and G6PD deficiency were comparable between children with a VOE alone and with ACS during the study period; however, 33 (94%) children with ACS had more severe genotypes (HbSS, HbSβ^0^, or HbSDPunjab), and 22 (62.8%) had a history of ACS. Children in both groups were equally treated with hydroxyurea. High steady-state leukocyte/neutrophil counts and high LDH level were significantly associated with increased incidence of ACS.

### 3.2. Risk Factors of Acute Chest Syndrome Complication and Predictive Score

On bivariate analyses, SpO2, RR, morphine use, pain localization, leukocyte, reticulocyte and neutrophil counts, and Hb, LDH, and AST levels at admission were associated with ACS development but not symptom duration before admission, temperature, HR, CRP level, platelet count, or fibrinogen, bilirubin, and HbF levels (Table 2).

Multivariate analyses retained FPS, neutrophil and reticulocyte counts, and pain localization as independently associated with ACS development (Table 3).

A score predicting ACS was calculated for the 149 VOEs (120 ACS and 29 non-ACS) with no missing data for any of the parameters included (Table 3). The mean score was 11.58 (±6.51) (range 0 to 23). The area under the ROC curve was 0.85 (95% CI = 0.79–0.92). On the basis of these thresholds, we defined the following three risk groups for ACS: (1) low risk: score < 11 (*n* = 43), NPV = 97.7% (1/43); (2) high risk: score > 20 (*n* = 6), PPV = 83.3% (5/6); and (3) intermediate risk: score 11–20 (*n* = 100), NPV = 77% (77/100) (Table 3).

### 3.3. Short-Term Outcome

The median delay from admission to the incident ACS was two days (IQR 2–3). Only two children were transferred to the intensive care unit for non-invasive ventilation (length of treatment, five and six days). No child required intubation. In accordance with French national guidelines, all children with ACS received RBC transfusion at a median time of two days (IQR 1–3) after admission. A total of 28 (17.9%) children in the VOE group received RBC transfusion at a median time of one day (IQR 1–2) after admission. The median length of hospitalization was significantly longer with ACS than VOE alone (seven (IQR 6–9) vs three (2–4) days, *p* < 0.0001). We found no cases of stroke, invasive bacterial infection, acute splenic sequestration, or death.

### 3.4. Evolution of Clinical and Biological Parameters

**Figure 1:**
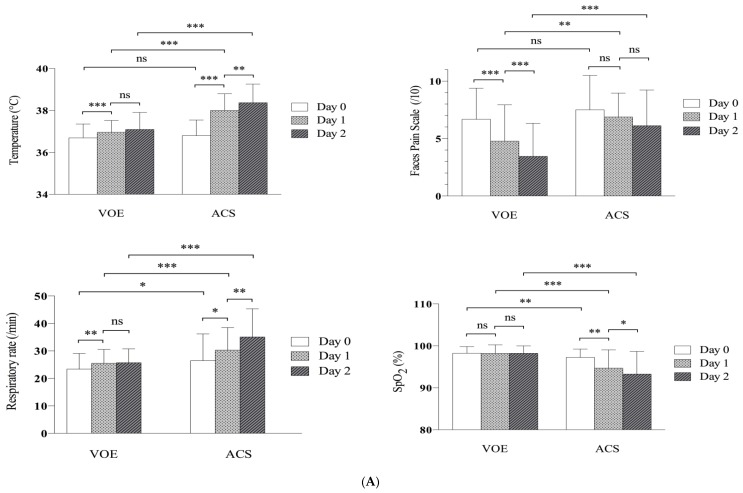

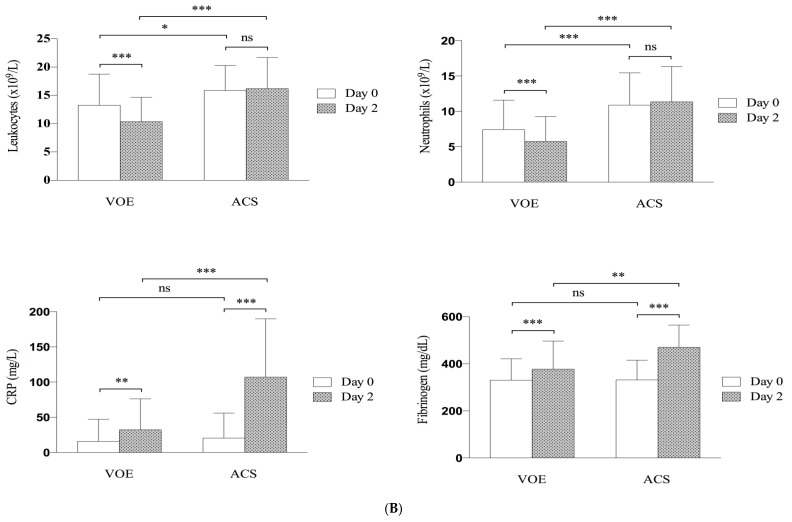

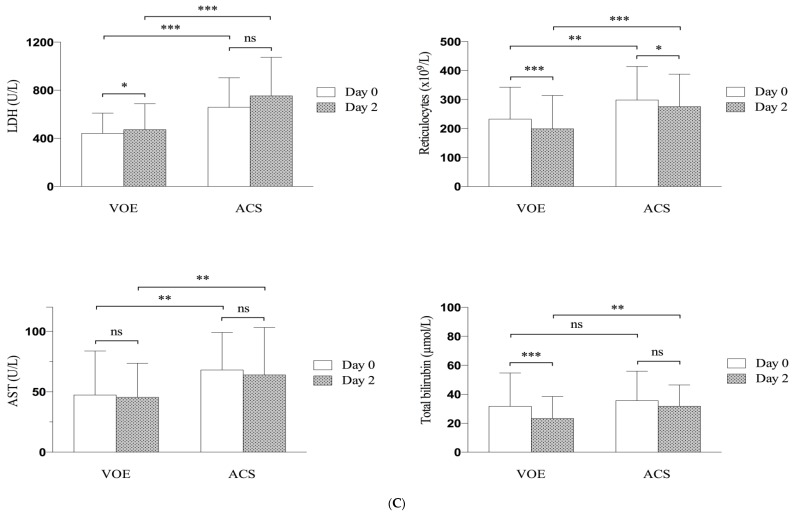
Dynamics of (**A**) clinical parameters (**B**) laboratory data in VOE versus ACS group and (**C**) hemolytic variables in VOE versus ACS group, by evolution of VOE in hospitalized children with sickle cell disease. VOE: vaso-occlusive episode; ACS: acute chest syndrome. * *p* < 0.05, ** *p* < 0.01, ***p < 0.001, ns: not significant. FPS, face pain score; SpO2, peripheral capillary oxygen saturation; CRP, C-reactive protein; LDH, lactate dehydrogenase; AST, aspartate aminotransferase.

Considering the dynamics of clinical and biological parameters, we found differences between ACS and VOE groups (Figure 1).

As expected, in the ACS group, clinical signs of lung injury developed over time, with decreased SpO2 level and increased RR and temperature (day 1 and 2). The pain score plateaued between day 0 and day 1 in the ACS group (*p* = 0.12), whereas the VOE group showed a significant improvement, as soon as day 1 (*p* < 0.001). At day 2, mean pain scores were significantly higher with ACS than VOEs alone (6.12 (±3.11) vs. 3.46 (±2.88), *p* = 0.0002) (Figure 1A).

Moreover, leukocyte and neutrophil counts plateaued from day 0 to 2 in the ACS group as compared with a significant reduction in the VOE alone group. At day 2, leukocyte count was significantly higher in the ACS versus VOE alone group (16.20 (±5.50) vs. 10.35 (±4.33), *p* < 0.0001).

CRP and fibrinogen levels increased in both groups from day 0 to day 2 but were significantly higher in the ACS than VOE alone group on day 2 (Figure 1B).

Hemolytic parameters (LDH and AST levels and reticulocyte count) were significantly increased in the ACS group versus VOE alone group, both at admission and on day 2, whereas total bilirubin level became significantly higher on day 2 only (Figure 1C).

## 4. Discussion

Several prospective and retrospective studies have attempted to identify risk factors associated with ACS development and recurrence [3,4,5,8]. In agreement with these studies, we found a high ACS incidence in children with more severe sickle genotypes or a history of ACS and those with high steady-state leukocyte count, but history of asthma or baseline fetal Hb level were not informative. Several reasons may account for these discrepancies. First, the main studies identifying risk factors associated with increased incidence of ACS analyzed the data on ACS episodes in general, regardless of the etiology [3,4], whereas our study focused on ACS episodes that developed over the clinical course of a VOE. Second, more than 40% of the children in our study were receiving disease-modifying hydroxyurea treatment, whereas previous studies were conducted before the introduction of hydroxyurea therapy.

Few studies have evaluated whether the clinical or laboratory features at initial diagnosis of a VOE are associated with secondary development of ACS [26,27,28]. We show that pain localization (abdominal or spinal pain or involving more than two limbs), pain score (≥9), and high reticulocyte (≥260 × 10^9^/L) and neutrophil (>10 × 10^9^/L) counts at admission were independently associated with evolution to ACS in children hospitalized for a painful VOE. Our results are highly consistent with those of a retrospective study assessing 175 children hospitalized for a VOE showing significantly high leukocyte count and low Hb level in those in whom ACS developed [26].

Our results support previous data in adults identifying high leukocyte count and spine pain as independently associated with ACS [27]. In contrast to the adult population, in children, abdominal pain is recurrent, and the management of an abdominal VOE is highly challenging in our experience. The multivariable model additionally retained reticulocyte count, which reflects hemolysis, in both studies. Both pediatric and adult studies support pathophysiological data suggesting an important role of leukocytes (neutrophils in particular) and hemolysis in ACS development [10,11,27]. High LDH level was the best outcome predictor in adults with SCD admitted for a VOE in another French study [28]. Although bivariate analyses identified LDH levels at admission, as associated with ACS development (LDH levels were higher for children with ACS versus VOEs alone (657.1 (±245.4) vs. 440.4 (±168.2), *p* < 0.001)), in vitro hemolysis (during and after blood withdrawal) precluded the measurement of LDH values in 48 of our cases. LDH level was therefore excluded in the multivariable analysis (only variables with < 20% missing values were entered in the model).

On bivariate analyses, the use of morphine at admission was strongly associated with ACS development. Other studies have suggested that opioid selection and morphine dose during a pain crisis may affect ACS development, the postulated mechanisms including hypoventilation and reduced mobilization, leading to atelectasis [26,29,30]. However, whether the choice of morphine specifically or the pain that is severe enough to require morphine is associated with ACS development remains to be determined. In the multivariable model for predicting the development of ACS, pain score was included rather than morphine use because it is more clinically relevant.

Although temperature did not differ and RR only slightly differed between ACS and VOE groups at admission, the difference gained clinical significance as soon as day 1 and further increased on day 2. SpO2 level was significantly lower at admission for children with than without ACS (97% vs. 99%), and further decreased over time (96% at day 1 and 93% at day 2). These observations confirm that early but subtle signs precede the diagnosis of ACS. Lung ultrasonography, which has been found sensitive and specific for the diagnosis of ACS in children admitted for fever, could be further investigated in the setting of children hospitalized for a VOE. Whether systematic daily screening might help to detect early signs of ACS needs to be demonstrated but would clearly allow for earlier therapeutic interventions [31].

Some studies have described significant changes from steady-state values at VOE onset (increase in leukocyte and reticulocyte counts and CRP level and decrease in Hb level) [32,33]. Our study is the first to describe the chronology over the first 48 h of both VOE and ACS in the setting of hospitalization for a VOE in children. We identified striking differences in the evolution profiles between groups: for children with VOE alone, pain score rapidly decreased from day 0 to day 1, then to day 2 but remained elevated during the same period for children with ACS. At day 2, hyperleucocytosis began to decrease in children with VOE alone but plateaued for children with ACS. CRP level for both groups increased on day 2 but only slightly in the VOE alone group. When RBC transfusion was considered during a VOE, but the indication is not clear-cut (in clinical practice, when the interpretability of the child-reported pain score is difficult), then the analysis of leukocyte count and CRP level at day 2 may help in a decision. Interestingly, a recent study performed in adult patients with severe ACS hospitalized in intensive care unit, comparing ACS-related lung ultrasound patterns at admission and after 48 h reported that the evolution of lung aeration during the first two days was strongly associated with outcome events occurring beyond this time point [34]. Early detection of patients who will experience a complicated outcome during VOE and ACS is a major concern, since it would allow physicians to modify their management accordingly.

The immediate aim of treatment for ACS is to prevent or reverse the acute respiratory failure, but treatment should also aim at reducing morbidity, including discomfort, pain, and length of hospitalization. Most experts recommend blood transfusions as first-line treatment because they can produce rapid improvements in clinical, radiological, and oxygenation parameters [4]. However, the increasing recognition of the risk of transfusion in patients with SCD has led to the development of alternative strategies such as early non-invasive ventilation (NIV), restricting transfusions to the most severe cases. Only one randomized study of intermittent NIV for ACS in adults has been reported, with no evidence of blood gas parameter improvements [35]. A recent pediatric study suggested that early NIV was well tolerated in children with ACS and might spare transfusion in some children [14]. In this previous study, only 23/66 (35%) children received RBC transfusions for ACS episodes, but the median hospital stay was 10 days as compared with 7 days in our study, systematic and early transfusion. Moreover, the length of hospital stay, calculated after the diagnosis of ACS in the former study would have been even longer if calculated from admission, which was the case in our study. Pain, hospital stay, and hypoxia duration may adversely affect the quality of life of children and caregivers. We believe that these are endpoints that should ideally drive medical decision-making.

In our study, 28 (17.9%) children with a VOE alone received early transfusion (at a median time from admission of one day [IQR 1–2]) and did not experience ACS. Children with ACS received erythrocyte transfusion at a median of two days [IQR 1–3]. This finding might argue for a beneficial effect of early RBC transfusion to prevent ACS development. However, when early-transfused VOE episodes were compared with ACS episodes, although clinically identical at admission (pain scores, temperature, SpO2, RR, HR), neutrophil counts differed significantly (7.36 (±4.76) vs. 10.89 (±4.55), *p* = 0.008). Because children with ACS differed biologically at admission from children with a VOE alone receiving erythrocyte transfusion, it seems unlikely that our results are biased by early erythrocyte transfusion during VOE.

Children with a VOE alone receiving RBC transfusion (*n* = 28) differed significantly from non-transfused children (*n* = 128), in terms of anemia severity (mean Hb level 8.1 (±1.7) vs. 9.2 (±1.4), *p* = 0.0001) and FPS at admission (7.6 (±2.9) vs. 6.4 (±2.6), *p* = 0.03). This finding is consistent with French guidelines indicating blood transfusion for significant anemia or severe and/or morphine-resistant VOE [23].

Because the aim of our study was to assist pediatricians in the early recognition of ACS complications during a VOE, to improve care for children with SCD, our study focused on risk factors present at admission, associated with further ACS development. We believe this is essential, considering that most acute complications in children with SCD are managed by emergency and general pediatric teams, not primarily involved in the care of children with SCD, and with no systematic access to medical records and medical history. Therefore, we developed a pediatric score based on four simple clinical parameters (pain localization (abdominal or spinal pain or involving more than two limbs) and pain score (≥9/10) as well as laboratory parameters (reticulocyte and neutrophil counts) at admission. Its application may help identify children at increased risk of ACS development during a VOE. For every child at intermediate and high risk of ACS, vigilance should be maintained throughout hospitalization, with regular monitoring of vital signs (HR, BP, RR, and SpO2 on air) and at least twice-daily chest examination.

Our study was homogenous because of its monocenter design, with the same physicians and guidelines for all patients. However, our study presents some limitations. First, this was a monocentric study, and second, we included a rather overall small number of children developing ACS during the course of VOE. Thus, the score for predicting ACS developed in our study remains to be validated in multi-center studies, before concluding as to its usefulness. Additionally, considering the gaps in preemptive treatments, our study was conducted as a preliminary step before assessing specific and early strategies to reduce ACS complication. We are currently evaluating the accuracy of lung ultrasonography to further predict ACS development in children admitted with a VOE. We suggest that the benefit of preemptive transfusion could be investigated in children admitted for high-risk VOC.

## 5. Conclusions

Identification of characteristics associated with ACS development in children with SCD hospitalized with VOE is crucial to better understand the pathophysiology of ACS but also to design interventions to decrease the morbidity of the disease. Our study identified laboratory (reticulocyte and neutrophil counts) and clinical parameters (pain score and pain localization) at hospital admission that were associated with the development of ACS in children admitted for a painful VOE. To assist pediatricians in the early recognition of ACS complications, we developed a simple score for predicting ACS from admission. In addition, our results suggest that evolution of leukocyte count and CRP level at day 2 could be a reliable criterion to define VOE severity. Finally, the score may help select children for future trials evaluating RBC transfusion or other treatment options for preventing ACS.

## Figures and Tables

**Table 1:** Patient history and laboratory parameters at steady state according to the development of acute chest syndrome (ACS) after hospitalization for a vaso-occlusive episode (VOE) in children with sickle cell disease.

Patient History	All Children *n* = 176	Children with VOEs Alone *n* = 141	Children with ACS *n* = 35	*p*
Age, mean ± SD (*n* = 141/35)	9.07 (±5.24)	9.05 (±5.40)	9.17 (±4.59)	0.89
Sex (*n* = 141/35)				
Female (*n*, %)	89 (50.57)	74 (52.48)	15 (42.86)	0.30
G6PD status (*n* = 132/33)				
Normal (*n*, %)	151 (91.52)	122 (92.42)	29 (87.88)	0.40
Deficiency (*n*, %)	14 (8.48)	10 (7.58)	4 (12.12)	
Genotype (*n* = 141/35)				
SC, Sβ^+^ (n, %)	33 (18.75)	31 (21.99)	2 (5.71)	**0.027**
SS, Sβ^0^, SDPunjab (*n*, %)	143 (81.25)	110 (78.01)	33 (94.29)	
History of asthma (*n* = 136/35)				
Presence (*n*, %)	19 (11.11)	13 (9.56)	6 (17.14)	0.20
History of ACS (*n* = 136/35)				
Presence (*n*, %)	81 (47.37)	59 (43.38)	22 (62.86)	**0.04**
Hydroxyurea treatment (*n* = 141/35)				
Yes	74 (42.05)	58 (41.3)	16 (45.71)	0.62
No	102 (57.95)	83 (58.87)	19 (54.29)	
Markers at steady state (Mean, SD)				
Hemoglobin, g/dL (*n* = 130/35)	8.78 (1.50)	8.91 (1.49)	8.30 (1.44)	**0.03**
Fetal hemoglobin, % (*n* = 128/33)	14.22 (8.93)	14.26 (9.22)	14.08 (7.86)	0.91
Leukocyte count, 10^9^/L (*n* = 129/35)	12.66 (5.10)	12.03 (4.68)	14.96 (5.94)	**0.002**
Neutrophil count, 10^9^/L (*n* = 125/35)	5.44 (3.21)	4.94 (2.59)	7.23 (4.40)	**0.0001**
Platelet count, 10^9^/L (*n* = 128/35)	346.926 (121.561)	337.968 (114.771)	379.685 (140.706)	0.071
Mean corpuscular volume, fl (*n* = 128/35)	74.22 (8.65)	73.87 (8.52)	75.52 (9.10)	0.31
Reticulocyte count, 10^9^/L (*n* = 128/35)	264.120 (126.255)	245.922 (121.912)	330.670 (120.954)	**0.0004**
Lactate dehydrogenase level, U/L (*n* = 108/32)	582.77 (325.69)	565.37 (333.48)	641.53 (295.23)	0.24
Total bilirubin level, µmol/L (*n* = 101/29)	25.92 (16.60)	24.78(16.15)	29.89(17.80)	0.14

Bold values indicate significance at *p* < 0.05. Quantitative data are shown as mean ± SD and were compared by Student *t* test, and categorical variables are shown as numbers (percent) and were compared by chi-square test. VOE: vaso-occlusive episode; ACS: acute chest syndrome; G6PD: glucose-6-phosphate dehydrogenase.

**Table 2:** Clinical and laboratory parameters at admission (day 0) by development or not of ACS during VOEs in children with sickle cell disease.

Clinical Variables	Episodes Limited to VOEs *n* = 156	Episodes with ACS During VOEs *n* = 35	*p*
Symptoms duration before admission, day	1.51 (±2.98)	0.85 (±1.16)	0.19
Temperature, °C	36.7 (±0.6)	36.8 (±0.7)	0.36
Heart rate, /min	103.4 (±22.8)	104.0 (±20.8)	0.88
Respiratory rate, /min	23.4 (±5.7)	26.4 (±9.7)	**0.046**
Oxygen saturation on air, %	98.2 (±1.6)	97.2 (±2.0)	**0.002**
Faces Pain Score, /10	6.7 (±2.7)	7.5 (±2.8)	0.14
Use of morphine, *n* (%)	20 (14.3)	15 (45.4)	**<0.001**
Pain localization, *n* (%)			
Abdominal	36 (23.1)	16 (45.7)	**0.007**
Thoracic	29 (18.8)	9 (25.7)	0.35
Spinal	36 (23.1)	16 (45.7)	**0.007**
Pain restricted to arms or legs (≤ 2 localizations) *	62 (39.7)	3 (8.6)	**<0.001**
Pain restricted to arms or legs (> 2 localizations)	9 (5.8)	2 (5.7)	1
**Laboratory Parameters**			
Leukocyte count, 10^9^/L	13.27 (±5.50)	15.86 (±4.43)	**0.01**
Neutrophil count, 10^9^/L	7.41 (±4.18)	10.89 (±4.55)	**<0.001**
Platelet count, 10^9^/L	341.892 (±123.215)	354.647 (±114.248)	0.58
Hemoglobin level, g/dL	9.0 (±1.5)	8.2 (±1.1)	**0.0031**
Reticulocyte count, 10^9^/L	232.295 (±110.571)	298.574 (±115.262)	**0.0029**
Alanine aminotransferase level, U/L	24.7 (±33.9)	28.1 (±24.5)	0.56
Aspartate aminotransferase level, U/L	47.4 (±36.4)	68.0 (±31.2)	**0.0038**
Total bilirubin level, µmol/L	31.7 (±22.9)	35.6 (±20.3)	0.36
Lactate dehydrogenase level, U/L	440.4 (±168.2)	657.1 (±245.4)	**<0.001**
C-reactive protein level, mg/L	15.8 (±31.4)	20.4 (±35.4)	0.45
Fibrinogen level, mg/dL	3.3 (±0.9)	3.3 (±0.8)	<0.78
Fetal hemoglobin, %	12.2 (±8.6)	9.7 (±4.3)	0.12

Data are mean (±SD) unless indicated. Bold values indicate significance at *p* < 0.05. VOE: vaso-occlusive episode; ACS: acute chest syndrome. Quantitative data are shown as mean ± SD and were compared by Student *t* test, and categorical data are shown as numbers (%) and were compared by chi-square test or Fisher exact test (*).

**Table 3:** Univariate and multivariable analyses and predictive model for acute ACS in children with sickle cell disease and hospitalized for a VOE.

Variable	Univariate Analysis			Multivariable Analysis			Score
	Crude OR ^a^	95%CI	*p* ^d^	aOR ^b^	95%CI	*p* ^d^	
Sex ^c^							
Male	1	–		1	–		
Female	0.69	0.33–1.46	*0.33*	0.41	0.15–1.10	*0.08*	
Faces Pain Score at day 0 ^c^							
<9	1	–		1	–		
≥9	3.13	1.40–6.96	*0.005*	3.65	1.37–9.75	***0.01***	4
Neutrophil count (10^9^/L) at day 0 ^c^							
≤10	1	–		1	–		
>10	4.00	1.85–8.66	*0.0004*	4.84	1.78–13.17	***0.002***	5
Reticulocytes count (10^9^/L) at day 0 ^c^							
<260	1	–		1	–		
≥260	2.35	1.07–5.16	*0.03*	3.07	1.14–8.30	***0.03***	3
Pain restricted to arms or legs (≤ 2 localizations) at day 0 ^c^							
Yes	1	–		1	–		
No	7.04	2.06–23.98	*0.002*	11.23	2.21–57.15	***0.004***	11
**Predictive score ^e^**	**VOEs with ACS**	**VOEs without ACS**	**Total**				
>20	5	1	6	PPV = 83.3%			High
11–20	23	77	100	NPV = 77%			Intermediate
<11	1	42	43	NPV = 97.7%			Low
Total	29	120	149				

Test de Hosmer-Lemeshow: chi-square = 4.14, *p* = 0.84. R^2^ Nagelkerke = 0.38. R^2^ Mac Fadden = 0.28. Area under the receiver operating characteristic curve: 0.85 (95% CI: 0.79–0.92, *p* < 0.0001). ^a^ Non-adjusted odds ratio (OR) > 1 indicates a risk factor for ACS. The link is significant if the value 1 is not included in the 95% CI. ^b^ Adjusted odds ratio (OR) > 1 indicates a risk factor for ACS. The link is significant if the value 1 is not included in the 95% CI. ^c^ Variables included in the multivariate model (*p* < 0.20). Variable sex was forced in the model. ^d^ *p* < 0.05 indicates that the candidate variable is associated with the ACS variable (Wald test). ^e^ Calculated by rounding the ORs for the above variables applicable to each patient. PPV, positive predictive value; NPV, negative predictive value.
